# Modulation of Triplet-State Reactivity and Enhanced Singlet Oxygen Generation in Tricyclic Thiopurine Analogues

**DOI:** 10.3390/ijms27125482

**Published:** 2026-06-17

**Authors:** Katarzyna Taras-Goslinska, Katarzyna Krancewicz, Bronislaw Marciniak

**Affiliations:** 1 Faculty of Chemistry, Adam Mickiewicz University, Uniwersytetu Poznanskiego 8, 61-614 Poznan, Poland; katarzyna.krancewicz@amu.edu.pl (K.K.); marcinia@amu.edu.pl (B.M.); 2 Centre for Advanced Technology, Adam Mickiewicz University, Uniwersytetu Poznanskiego 10, 61-614 Poznan, Poland

**Keywords:** thiopurines, triplet state, singlet oxygen

## Abstract

Thiopurines are efficient triplet-state photosensitisers; however, the practical application of canonical derivatives such as 6-thioguanine (6TG) and 6-thioguanosine (6TGuo) is limited by competing deactivation pathways that reduce the fraction of triplet states available for productive interaction with molecular oxygen. In this work, we investigated how structural modification of the thiopurine scaffold through introducing of an additional five-membered etheno ring affects triplet-state energetics, deactivation pathways, and singlet oxygen sensitisation. The photophysical properties of four tricyclic thiopurine analogues—9-thio-1,N^2^-ethenoguanine (TEGua), 9-thio-1,N^2^-ethenoguanosine (TEGuo), 6-methyl-9-thio-1,N^2^-ethenoguanine (6MeTEGua), and 6-methyl-9-thio-1,N^2^-ethenoguanosine (6MeTEGuo)—were investigated using steady-state spectroscopy, low-temperature phosphorescence, nanosecond transient absorption spectroscopy, and direct detection of singlet oxygen phosphorescence. All investigated compounds exhibited efficient intersystem crossing and microsecond-lived triplet states. Compared with canonical thiopurines, the tricyclic analogues displayed lower triplet-state energies and significantly enhanced singlet oxygen generation. Quantum yields of singlet oxygen sensitisation reached ~0.56 in acetonitrile, approximately twofold higher than those observed for 6TG and 6TGuo under identical conditions. Analysis of triplet-state deactivation pathways showed that the enhanced photosensitising efficiency does not result from increased triplet formation, but from more effective use of the triplet-state population for energy transfer to molecular oxygen leading to singlet oxygen formation. These findings demonstrate that structural modification of the thiopurine scaffold enables control over triplet-state reactivity and provides a strategy for designing improved thiopurine-based photosensitisers for photodynamic therapy applications (PDT).

## 1. Introduction

Thiopurines, including 6-thioguanine (6TG) and its nucleoside analogue 6-thioguanosine (6TGuo), are clinically important drugs widely used in cancer therapy, immunosuppressive treatments, and the management of rare diseases. Their biological activity primarily stems from their incorporation into nucleic acids, where they interfere with DNA replication and repair [1,2,3,4]. However, beyond their well-established pharmacological roles, thiopurines also exhibit distinct photochemical activities.

Thiopurines belong to a class of compounds in which the oxygen atom of the carbonyl group in guanine, hypoxanthine, and their nucleosides is replaced by a sulphur atom. This seemingly minor modification profoundly alters the spectral, photophysical, and photochemical properties of purine derivatives, opening the possibility of their use in photodynamic therapy (PDT). Upon UVA irradiation, 6TG and related compounds efficiently populate the lowest triplet excited state (T_1_) via intersystem crossing (ISC), a process strongly facilitated by the sulphur atom through enhanced spin–orbit coupling [5,6,7]. As a result, the quantum yield of triplet formation approaches unity, making thiopurines among the most efficient triplet generators in biologically relevant systems. Extensive spectroscopic studies have characterised the excited-state dynamics of 6TG and its derivatives, revealing triplet states with microsecond lifetimes and multiple deactivation pathways, including non-radiative decay, self-quenching, and interactions with surrounding molecules [8,9,10,11,12].

Of particular importance is thiopurine’s ability to sensitise molecular oxygen. Energy transfer from the triplet state to ground-state oxygen produces singlet oxygen (^1^O_2_), a highly reactive species that causes oxidative damage to biomolecules [13,14,15,16,17]. This process is directly linked to the phototoxicity of thiopurine incorporated DNA, in which UVA irradiation induces oxidative lesions and mutagenesis [18,19,20,21]. These properties have also motivated the investigation of thiopurines as potential photosensitisers for PDT, a treatment modality that relies on the controlled generation of reactive oxygen species to selectively destroy diseased cells [22,23,24].

Despite their favourable photophysical properties, classical thiopurines have limited applicability in PDT because of their low selectivity. In complex biological environments, the triplet states of thiopurines are efficiently quenched by nucleic acids and other biomolecules [11,12,21,22,25]. These interactions reduce the fraction of triplet states available for productive oxygen sensitisation and promote competing pathways, thereby limiting their therapeutic potential. Consequently, the key challenge in designing thiopurine-based photosensitisers is not to enhance triplet formation, which is already highly efficient, but to control the fate of the triplet state and direct it towards the desired photochemical pathways.

In this context, structural modification of the thiopurine scaffold is a promising strategy to harness triplet-state reactivity. In particular, extending the π-conjugated system via introducing an additional ring is expected to affect both the energy and reactivity of the T_1_ state, potentially enabling more efficient interactions with molecular oxygen while suppressing competing deactivation pathways.

In our previous studies [26], we reported the synthesis of a new class of tricyclic etheno-thiopurine analogues, together with their biological activity and photochemical reactivity, including the identification of stable photoproducts. These findings demonstrate that modifications to the thiopurine core lead to significant changes in photochemical behaviour. However, the mechanistic origin of these differences, particularly in excited-state dynamics, remains to be fully elucidated.

In this work, we focus on the detailed characterisation of the lowest triplet excited state, T_1_, which plays a central role in thiopurine photochemistry. We investigate the formation, energy, and deactivation pathways of T_1_ in tricyclic thiopurine analogues and compare their properties directly with those of the parent compounds, 6TG and 6TGuo.

We demonstrate that, although both systems exhibit near-unity triplet yields, the tricyclic analogues generate singlet oxygen with significantly higher efficiency. This enhancement arises not from increased triplet formation but from more effective utilisation of the triplet state, resulting from a lower triplet-state energy and a more favourable distribution of deactivation pathways.

These findings provide novel insights into the relationship between molecular structure and triplet-state reactivity. More importantly, they show that the key parameter governing photosensitiser performance is not the efficiency of triplet formation itself, but rather the extent to which the triplet state is channelled into productive photochemical processes. This concept provides a framework for the rational design of thiopurine-based photosensitisers with improved selectivity and potential applications in photodynamic therapy (PDT).

## 2. Results and Discussion

### 2.1. Spectral Characteristics

**Figure 1:**
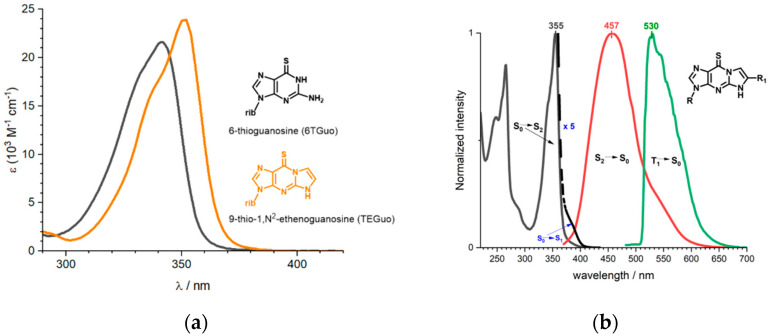
(**a**) Absorption spectra of 6-thioguanosine (6TGuo) and tricyclic thiopurine analogue (TEGuo) in aqueous solution. (**b**) Relative positions of absorption, fluorescence recorded in CCl4, and phosphorescence spectra of a model representative tricyclic analogue (where R = β-D-ribose or R = H, R_1_ = CH_3_ or R_1_ = H) recorded in a glassy matrix (MeOH:CH_2_Cl_2_, 1:1) at 77 K.

The investigated 1,N^2^-etheno thiopurine analogues, namely 9-thio-1,N^2^-ethenoguanine (TEGua), 9-thio-1,N^2^-ethenoguanosine (TEGuo), 6-methyl-9-thio-1,N^2^-ethenoguanine (6MeTEGua), and 6-methyl-9-thio-1,N^2^-ethenoguanosine (6MeTEGuo), are derived from the 6-thioguanine/6-thioguanosine scaffold by introducing an additional five-membered etheno ring, thereby extending the π-system while preserving the thiocarbonyl chromophore. Compared with 6TG/6TGuo, which show a dominant absorption maximum at ~340 nm, the tricyclic analogues display a bathochromically shifted and broadened low-energy band extending beyond 350 nm (Figure 1a), indicating that ring annulation lowers the transition energy without reducing its intensity. The intense band in the 320–400 nm region (ε > 2 × 10^4^ M^−1^ cm^−1^) is assigned to an allowed π → π* (S_0_ → S_2_) transition, while a weak long-wavelength band (~400 nm, ε ≈ 250 M^−1^ cm^−1^) corresponds to the forbidden n → π* (S_0_ → S_1_) transition characteristic of thiocarbonyl systems (Figure 1b) [11,27]. Consistent with our previous studies, fluorescence is weak (Φ_F_ ~ 10^−3^) and originates from the S_2_ state, with no detectable emission from S_1_ [26]. This behaviour indicates highly efficient non-radiative deactivation, particularly intersystem crossing, identifying the triplet-state as the dominant relaxation pathway. To directly assess triplet-state energetics, phosphorescence spectra were recorded in a glassy matrix (MeOH:CH_2_Cl_2_, 1:1) at 77 K under identical conditions for all tricyclic analogues and the reference compounds (6TG and 6TGuo). All tricyclic derivatives exhibit phosphorescence between 510 and 700 nm, with maxima around 530 nm (Figure 1b). The corresponding triplet-state energies (E_T_ ≈ 18,800–19,100 cm^−1^) are systematically lower than those determined for 6TG and 6TGuo (E_T_ ≈ 20,580 cm^−1^ under identical experimental conditions). The observation of phosphorescence at 77 K confirms efficient population of the T_1_ state upon UVA excitation. Although the tricyclic analogues exhibit triplet state energies approximately 1500–2000 cm^−1^ lower than those of 6TG and 6TGuo, both groups retain sufficient triplet-state energy to efficiently sensitise singlet oxygen.

### 2.2. Characterisation and Deactivation Pathways of the Lowest Triplet Excited State T_1_

#### 2.2.1. Characterisation of the Triplet State (T_1_) in the Absence of Oxygen

**Figure 2:**
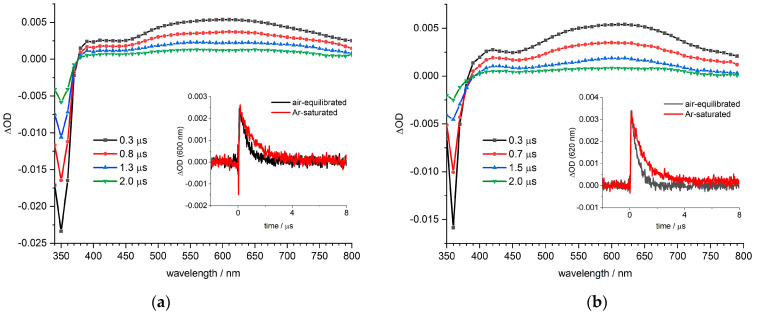
Transient absorption spectra of (**a**) TEGuo (1.3 × 10^−5^ M) and (**b**) 6MeTEGuo (1.3 × 10^−5^ M) in Ar-saturated aqueous solution at different delay times after the laser pulse. Inset: triplet-state decay traces measured for (**a**) TEGuo (1.3 × 10^−5^ M) and (**b**) 6MeTEGuo (1.3 × 10^−5^ M), monitored at the maximum of the transient absorption spectrum (λ = 650 nm) in Ar-saturated (red line) and air-equilibrated (black line) aqueous solution.

**Figure 3:**
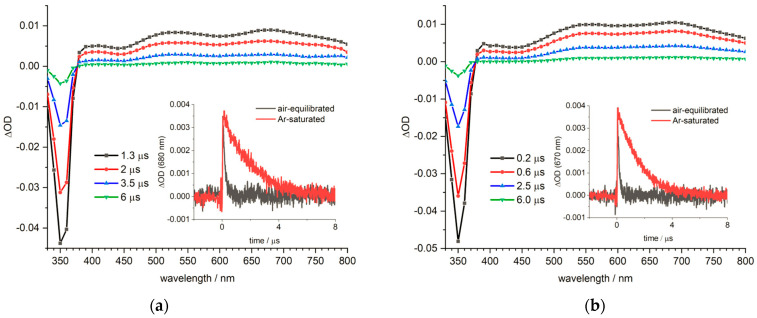
Transient absorption spectra of (**a**) TEGuo (1.3 × 10^−5^ M) and (**b**) 6MeTEGuo (1.3 × 10^−5^ M) in Ar-saturated acetonitrile at different delay times after the laser pulse. Inset: triplet-state decay traces measured for (**a**) TEGuo (1.3 × 10^−5^ M) and (**b**) 6MeTEGuo (1.3 × 10^−5^ M) monitored at the maximum of the transient absorption spectrum (λ = 680 nm) in Ar-saturated (red line) and air-equilibrated (black line) acetonitrile.

Spectral and kinetic data for the excited states of TEGuo, TEGua, 6MeTEGuo, and 6MeTEGua were obtained by nanosecond transient absorption spectroscopy. Upon excitation at 355 nm, all compounds formed a transient species that decayed on the microsecond timescale, consistent with the lowest triplet excited state. Figure 2 shows representative transient absorption spectra recorded in Ar-saturated H_2_O, and Figure 3 shows those in Ar-saturated ACN at various delay times after 355 nm laser excitation. The negative signal observed at λ < 360 nm is attributed to ground-state depopulation (bleaching). In the 380–800 nm range, the transient absorption decays with first-order kinetics, and the decay rate is independent of the monitoring wavelength, indicating that the entire spectrum can be assigned to a single excited species. The absorption intensity decreases in the presence of molecular oxygen. Although the spectral shape remains unchanged, the transient decays significantly faster under aerated conditions (Figure 2), indicating efficient quenching by oxygen. In addition, the decay rate increases linearly with solute concentration, indicating a contribution from bimolecular deactivation processes. The decay kinetics of the transient species were monitored at wavelengths corresponding to the maxima of the transient absorption bands. Across all investigated tricyclic thiopurines, the decay traces were monoexponential and independent of the monitoring wavelength, confirming the presence of a single transient species. Triplet lifetimes (τ_T_) were determined in both oxygen free water and acetonitrile solutions at comparable concentrations (c = 1.3 × 10^−5^ M). For the nucleoside derivatives, TEGuo and 6MeTEGuo, the lifetimes were 1.81 μs and 1.73 μs in acetonitrile, and 1.01 μs and 0.85 μs in water, respectively. The corresponding nucleobases, TEGua and 6MeTEGua, exhibited longer lifetimes of 2.45 μs and 2.37 μs in acetonitrile, and 1.42 μs and 1.21 μs in water (see Supplementary Materials). The assignment of the transient to the T_1_ state is supported by its microsecond lifetime and oxygen sensitivity. Importantly, the transient absorption spectra are identical for nucleobases and their corresponding nucleosides, as well as for methylated and non-methylated derivatives, indicating that substitution at C-(6) and glycosylation do not significantly affect the spectral characteristics of the triplet state.

The decay kinetics of the transient species were further analysed to quantify the deactivation pathways of the triplet state. The decay traces are monoexponential and independent of the monitoring wavelength, confirming the presence of a single transient species. The triplet lifetimes (τ_T_) are in the microsecond range and depend on both solvent and molecular structure, with nucleobases exhibiting longer lifetimes than nucleosides and acetonitrile yielding longer lifetimes than water.

**Figure 4:**
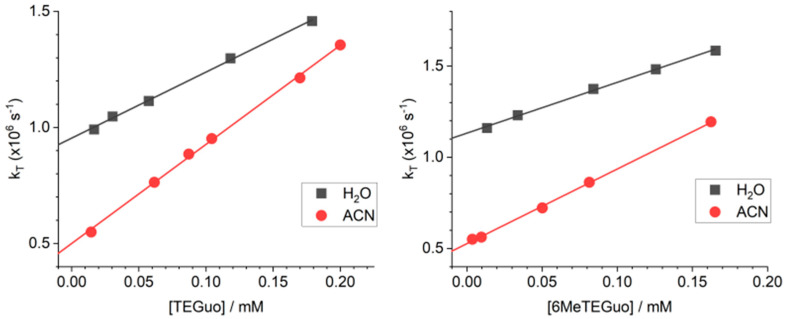
The rate of triplet-state decay (k_T_) of TEGuo/6MeTEGuo against the ground-state concentration under Ar-saturated conditions in ACN and aqueous solution.

A concentration-dependent shortening of the triplet lifetime was observed for all compounds, indicating the occurrence of self-quenching processes. The concentration-independent, intrinsic lifetimes τ_0_ and the self-quenching rate constants (k_sq_) were obtained from the intercept and slope, respectively, of the Stern–Volmer plots (Figure 4), and the values are shown in Table 1.

Based on these lifetimes, their shortening in the presence of oxygen, and their dependence on solute concentration, the observed transient species is assigned to the lowest triplet excited state (T_1_), consistent with previous studies of thiocarbonyl-containing systems [11,27,28]. Importantly, the triplet lifetime decreases with increasing solute concentration across all investigated compounds.

To obtain kinetic information on the intramolecular decay of the excited triplet state, the rate constants for radiative (k_P_) and nonradiative (Σk_nr_) were calculated using Equations (1)–(3), and the results are summarised in Table 1.

(1) $$\Phi_{T}=\Phi_{P}^{0}+\Phi_{R}+\Phi_{\mathrm{nr}}$$

(2) $$k_{P}=\Phi_{P}^{0}/\left(\tau_{T}^{0}\times \Phi_{T}\right)$$

(3) $$k_{\mathrm{nr}}=\Phi_{\mathrm{nr}}/\left(\tau_{T}^{0}\times \Phi_{T}\right)$$

Photochemical decay appeared to be a negligible channel of deactivation under irradiation of dilute (c ~ 10^−4^ M) solutions of in Ar-saturated ACN and aqueous solution (Φ_R_ ≤ 10^−4^, Table 1). This indicates that the compounds studied are photochemically stable in the absence of O_2_.

All investigated analogues of tricyclic thiopurine exhibit near-unity triplet quantum yields, indicating that intersystem crossing is the dominant deactivation pathway for the excited singlet states (Table 1). It should be emphasised that, for previously studied thiopurines, the triplet-state formation quantum yield was also close to unity [6,11].

Such an efficient process is characteristic of sulphur containing chromophores, in which strong spin–orbit coupling facilitates efficient singlet triplet mixing [11,27].

The photophysical parameters of the lowest excited triplet states of TEGuo and 6MeTEGuo under anaerobic conditions are summarised in Table 1 (detailed measurement procedures are described in Section 3). The parameters for TEGua and 6MeTEGua are provided in the Supplementary Materials (Table S1).

The analysis of photophysical parameters shows that non-radiative decay is the dominant deactivation pathway in the absence of oxygen, with Φ_nr_ ≈ 0.90 and largely independent of solvent. These values are comparable to those reported for related thiopurines, such as thioinosine (Φ_nr_ > 0.99 in acetonitrile) [11].

#### 2.2.2. Interaction with Oxygen and Singlet Oxygen Generation

The purpose of our work was to investigate how altering the structure of thiopurines by introducing an additional five-membered ring affects the efficiency of processes occurring in excited states. In particular, our focus was on whether a group of tricyclic thiopurine analogues generates singlet oxygen and on determining the quantum yield of that generation. A high quantum yield for this process would provide a basis for further studies to confirm the potential of TEGuo and TEG for photodynamic therapy.

It should be emphasised that, for previously studied thiopurines, the quantum yield of triplet-state formation was also close to unity, which confirms that, for compounds containing a thiocarbonyl group in their structure, the triplet state is the main deactivation channel for excited states.

It is known that oxygen molecules effectively quench the T_1_ triplet excited state of thiocarbonyl compounds [11,27,28,29]. This prompted us to investigate the properties of the T_1_ triplet states of TEGuo, 6MeTEGuo, TEGua, and 6MeTEGua in the presence of oxygen (O_2_ in air-saturated solutions) in water and acetonitrile. It was observed that the triplet lifetime of the studied compounds in acetonitrile (c = 1.3 × 10^−5^ M) in air is approximately 14 times shorter than in an argon-saturated solution. For the non-deoxygenated sample in water (c = 1.3 × 10^−5^ M), a twofold reduction in lifetime was observed compared with the sample studied in an argon atmosphere. The observed quenching is greater in acetonitrile than in water, reflecting the difference in oxygen solubility (and thus oxygen concentration) between the two solvents. The oxygen concentration in ACN is nearly ten times higher than in water ($c_{O_{2}}^{ACN}(0.21\;atm)=$ 1.9 × 10^−3^ M; $c_{O_{2}}^{H_{2}O}$(0.21 atm) = 2.7 × 10^−4^ M) [30].

The triplet lifetimes, τ_T_, measured under aerated and deoxygenated conditions, were used to estimate the rate constant for quenching by molecular oxygen (k_q_). The values were calculated using Equation (4), which accounts for self-quenching (k_sq_).

(4) $$\frac{1}{\tau_{obs}}=\frac{1}{\tau_{T}}+k_{q}\left[O_{2}\right]+k_{sq}c$$

where $\tau_{obs}$—lifetime of the triplet state in the sample in air; $\tau_{T}$—lifetime of the triplet state in the deoxygenated sample; $\;k_{q}$—rate constant for the quenching of the triplet sate by molecular oxygen; $\left[O_{2}\right]$—oxygen concentration in the solvent in which the measurements were carried out; $k_{sq}$—the self-quenching constant for the investigation compound; and c—the concentration of compounds under investigation.

The k_q_ values for TEGuo and 6MeTEGuo in both water and acetonitrile are on the order of 10^9^ M^−1^ s^−1^ (Table 2), indicating diffusion controlled quenching, consistent with data reported for other thiocarbonyl nucleobase analogues. This confirms that the triplet states of the investigated compounds interact efficiently with molecular oxygen.

Confirmation that the T_1_ triplet state of tricyclic thiopurine analogues is efficiently quenched by molecular oxygen prompted us to investigate whether TEGua, TEGuo, 6MeTEGua, and 6MeTEGuo can act as sensitizers of singlet oxygen ^1^O_2_. One of the primary mechanisms of triplet state quenching by oxygen involves energy transfer from the excited triplet state of the sensitizer to ground-state molecular oxygen, leading to the formation of highly reactive ^1^O_2_ [31]. Singlet oxygen was detected directly by monitoring its characteristic phosphorescence emission in the near-infrared region (λ_max_ = 1270 nm). The presence of ^1^O_2_ was confirmed for all investigated compounds in both heavy water (D_2_O) and acetonitrile, demonstrating that the studied tricyclic thiopurine analogues act as singlet oxygen sensitisers. To quantify this process, the quantum yield for singlet oxygen generation (Φ_Δ_) was measured.

Across all compounds, Φ_Δ_ values are approximately 0.54–0.56 in acetonitrile and about half that in D_2_O (Table 2). This decrease can be attributed, among other factors, to differences in oxygen solubility between the solvents and to shorter triplet-state lifetimes in aqueous media compared with acetonitrile. To evaluate the effect of structural modification on singlet oxygen sensitisation efficiency, analogous measurements were performed on the parent compounds 6TG and 6TGuo under identical experimental conditions. The results show that TEGua, TEGuo, 6MeTEGua, and 6MeTEGuo generate singlet oxygen with nearly twice the efficiency of 6TG and 6TGuo (Table S2 and Figure S1, Supplementary Materials). The quantum yields determined for 6TG are 0.32 in acetonitrile and 0.20 in D_2_O, while for 6TGuo they are 0.27 and 0.15, respectively.

An important factor determining the efficiency of singlet oxygen sensitisation is the fraction of the triplet-state population that transfers energy to molecular oxygen (O_2_), leading to ^1^O_2_, denoted S_Δ_. This parameter is calculated from the previously determined rate constants (k_0_, k_sq_, and k_q_, see Section 3).

These results highlight the importance of triplet state utilisation, rather than its formation, in determining the efficiency of singlet oxygen generation.

#### 2.2.3. Comparison of Triplet-State Reactivity in Canonical Thiopurines and Tricyclic Derivatives

A comparison of the photophysical behaviour of canonical thiopurines (6TG/6TGuo) and their tricyclic analogues shows that, despite similarly high efficiencies of triplet-state formation, significant differences in triplet-state reactivity emerge. In both systems, intersystem crossing is highly efficient; however, the tricyclic analogues exhibit lower triplet-state energies and a greater fraction of the triplet population that participates in energy transfer to molecular oxygen, leading to singlet oxygen generation, as reflected in higher S_Δ_ values (Table 2). A comparison of S_Δ_ values shows a clear difference between the parent compounds and the tricyclic analogues. For 6TGuo, S_Δ_ is 0.32, whereas TEGuo (S_Δ_ = 0.62) and 6MeTEGuo (S_Δ_ = 0.67) show higher values in acetonitrile. These results indicate that, for the tricyclic thiopurine analogues, a larger fraction of the triplet-state population participates in energy transfer to oxygen that results in singlet oxygen formation.

For canonical thiopurines, a larger proportion of the triplet-state population is deactivated via competing non-productive pathways, limiting their efficiency as photosensitisers. In contrast, the tricyclic analogues more effectively channel the triplet-state population towards interaction with molecular oxygen, resulting in significantly enhanced singlet oxygen generation.

Importantly, as demonstrated in our previous studies, the triplet state of TEGuo and related tricyclic thiopurine analogues is not quenched by naturally occurring nucleosides under oxygen-free conditions [32]. This behaviour contrasts with that of canonical thiopurines, where interactions with nucleic acid components constitute an additional deactivation pathway. The absence of nucleoside quenching removes a major competing channel, allowing a larger fraction of the triplet-state population to remain available for productive interaction with molecular oxygen.

Taken together, these results demonstrate that the key factor governing photosensitisation efficiency is not the formation of the triplet state itself, but rather its effective utilisation. Structural modification of the thiopurine scaffold through introducing an additional ring enables control over triplet-state deactivation pathways, thereby improving singlet oxygen generation and enhancing the potential for photodynamic applications.

**Figure 5:**
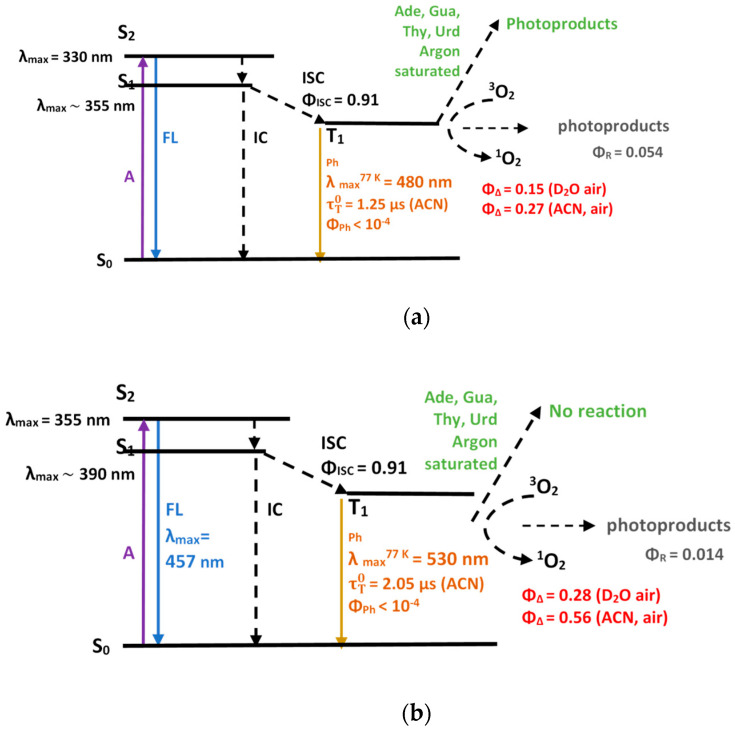
Schematic representation of excited-state pathways in (**a**) canonical thiopurines (6TG/6TGuo) and (**b**) tricyclic thiopurine analogues. (Solid arrows represent radiative processes, while dashed arrows represent non-radiative processes).

Schematic representations of excited-state pathways in (a) 6TG and (b) tricyclic thiopurine analogues are presented in Figure 5. While both systems exhibit efficient intersystem crossing, the tricyclic analogues show enhanced utilisation of the triplet state for singlet oxygen generation and reduced quenching by nucleosides.

## 3. Materials and Methods

### 3.1. Chemicals

All reagents and solvents were obtained from Sigma-Aldrich Chemical Co. (St. Louis, MO, USA) and from Merck (Darmstadt, Germany). Water was purified using a Milli-Q system (Millipore, Bedford, MA, USA).

The compounds described in this work were synthesized in our laboratory. The detailed synthesis and identification of the compounds were reported in a previous work [26].

### 3.2. Absorption and Emission Measurements

UV-vis absorption spectra were recorded using a Cary 100 and a Cary 300 Bio-Varian spectrophotometer (Agilent Technologies, Santa Clara, CA, USA), scanning from 800 to 200 nm with 1 nm increments in 1 cm × 1 cm quartz cells. Fluorescence spectra were recorded at room temperature in 1 cm × 1 cm quartz cells on a JASCO FP-8300 spectrofluorometer (Japan Spectroscopic Co., Ltd., Tokyo, Japan) (excitation and emission slits 5 nm, scan speed 500 nm min^−1^) for solutions with absorbance at the excitation wavelength less than 0.1.

### 3.3. Determination of Triplet–Triplet Molar Absorption Coefficients and Intersystem Crossing Quantum Yields

Triplet–triplet molar absorption coefficients (ε_T_) were determined using the singlet depletion method described previously [33]. The intersystem crossing quantum yields (Φ_ISC_) were determined relative to benzophenone used as a standard (ε_T_ = 6500 M^−1^ cm^−1^ at 520 nm). Solutions of the investigated compounds and the reference standard were prepared in the same solvent. Prior to measurements, the samples were deoxygenated. Transient absorption decay measurements were performed at wavelengths corresponding to the maxima of the transient absorption bands of the investigated compounds; for benzophenone, measurements were carried out at 520 nm. The ΔA values obtained for the investigated compounds and the reference were used to calculate Φ_ISC_ according to Equation (5):

(5) $$\Phi_{ISC}=\Phi_{ISC}^{ref}\frac{\varepsilon_{T}^{ref}}{\varepsilon_{T}}\frac{\Delta A}{\Delta A^{ref}}$$

where Φ_ISC_ is the intersystem crossing quantum yield of the investigated compound, $\Phi_{ISC}^{ref}$ is the intersystem crossing quantum yield of the reference compound, $\varepsilon_{T}^{ref}$ is the triplet–triplet molar absorption coefficient of the reference compound at the corresponding wavelength, ε_T_ is the triplet–triplet molar absorption coefficient of the investigated compound, ΔA is the transient absorption signal of the investigated compound, and ΔA^ref^ is the transient absorption signal of the reference compound.

### 3.4. Determination of Concentration-Independent Triplet Lifetimes, Self-Quenching Rate Constants, and Oxygen Quenching Rate Constants

The concentration-independent triplet-state lifetimes ($\tau_{T}^{0}$) were determined from transient absorption decay measurements performed over the concentration range 0.01–0.20 mM. Samples were deoxygenated for 20 min prior to measurements. Measurements were carried out in 1 cm quartz cuvettes at wavelengths corresponding to the maxima of the transient absorption bands.

The triplet lifetime (1/τ_T_) was plotted as a function of solute concentration. The $\tau_{T}^{0}$ values were obtained from the intercept of the linear fit with the y-axis. Self-quenching rate constants (k_sq_) were subsequently determined from the slope of the Stern–Volmer plots according to Equation (6):

(6) $$\frac{1}{\tau_{T}}=\frac{1}{\tau_{T}^{0}}+k_{sq}c$$

where τ_T_ is the experimentally measured triplet lifetime, $\tau_{T}^{0}$ is the concentration-independent triplet lifetime, k_sq_ is the triplet self-quenching rate constant, and c is the concentration of the investigated compound.

The rate constants for quenching of the triplet state by molecular oxygen [O_2_] (k_q_) were determined from transient absorption measurements performed under argon and air atmospheres. Solutions with concentrations on the order of 10^−5^ M were used to minimise the contribution of self-quenching processes. Each sample was divided into two equal portions: one was deoxygenated for 20 min before measurements, whereas the second was measured under air-saturated conditions immediately after preparation. Measurements were performed in 1 cm quartz cuvettes at wavelengths corresponding to the maxima of transient absorption bands. The oxygen quenching rate constants were calculated according to Equation (4).

### 3.5. Determination of Radiative and Non-Radiative Rate Constants

Radiative (k_p_) and non-radiative (k_nr_) rate constants were calculated using experimentally determined values of Φ_P_, Φ_R_, $\tau_{T}^{0}$, and Φ_ISC_ according to Equations (7)–(9):

(7) $$k_{p}=\frac{\Phi_{P}^{0}}{\tau_{T}^{0}\Phi_{ISC}}$$

(8) $$\Phi_{nr}=\Phi_{ISC}-(\Phi_{P}^{0}+\Phi_{R})$$

(9) $$k_{nr}=\frac{\Phi_{nr}}{\tau_{T}^{0}\Phi_{ISC}}$$

where $\Phi_{P}^{0}$ is the phosphorescence quantum yield at infinite dilution, $\tau_{T}^{0}$ is the concentration-independent triplet lifetime, Φ_ISC_ is the intersystem crossing quantum yield, and Φ_R_ is the quantum yield of the photochemical reaction under argon atmosphere.

### 3.6. Determination of Parameters Describing Triplet-State Reactivity Toward Molecular Oxygen

The fraction of the triplet-state population quenched by molecular oxygen and involved in singlet oxygen generation (S_Δ_) was calculated according to Equation (10):

(10) $$\Phi_{\Delta }=S_{\Delta }\Phi_{ISC}\frac{k_{q}[O_{2}]}{k_{0}+k_{sq}c+k_{q}[O_{2}]}$$

where Φ_Δ_ is the singlet oxygen quantum yield, Φ_ISC_ is the triplet-state formation quantum yield, k_q_ is the oxygen quenching rate constant, k_0_ is the concentration-independent triplet decay rate constant, k_sq_ is the self-quenching rate constant, and c is the concentration of the investigated compound.

### 3.7. Low-Temperature Emission Measurements

Emission and excitation spectra at 77 K were recorded using a JASCO FP-8300 spectrofluorometer. Measurements were performed in a glassy MeOH:CH_2_Cl_2_ (1:1, *v*/*v*) solvent mixture. Measurement parameters, including excitation and emission slit widths, detector sensitivity, chopping period, delay time, response time, and integration time, were optimised experimentally for each sample.

### 3.8. Determination of Singlet Oxygen Quantum Yields

Singlet oxygen quantum yields were determined by direct detection of singlet oxygen phosphorescence emission in the near-infrared region. Measurements were performed at room temperature in air-saturated solutions.

Singlet oxygen phosphorescence spectra were recorded using a FluoTime 300 fluorescence spectrometer (PicoQuant, Berlin, Germany) equipped with an NIR PMT H10330-45 detector (Hamamatsu Photonics K.K., Hamamatsu, Japan). Excitation sources included a xenon lamp (λ_exc_ = 352 nm) and laser diodes operating at λ_exc_ = 410 nm and 378 nm. Perinaphthenone was used as a reference standard (Φ_Δ_ = 0.95 ± 0.05, independent of solvent) [34]. Singlet oxygen quantum yields were determined using two methods.

### 3.9. Single-Point Method

Solutions of the investigated compounds and the reference standard were prepared in the same solvent. The absorbance values of the investigated compounds and the reference were matched within ±0.005 at the excitation wavelength. Singlet oxygen emission spectra were recorded, and the integrated emission intensities were used to calculate Φ_Δ_ according to Equation (11):

(11) $$\Phi_{x}=\Phi_{st}\frac{S_{x}}{S_{st}}\frac{\left(1-10^{-A_{st}}\right)}{\left(1-10^{-A_{x}}\right)}$$

where Φ_x_ is the singlet oxygen quantum yield of the investigated compound, Φ_st_ is the singlet oxygen quantum yield of the reference standard, S_x_ and S_st_ are the integrated singlet oxygen emission intensities for the investigated compound and the reference, respectively, and A_x_ and A_st_ are the absorbance values at the excitation wavelength.

### 3.10. Gradient Method

Solutions of the investigated compounds and the reference standard were prepared at three different concentrations in the same solvent. Absorbance values at the excitation wavelength were measured for each solution. Singlet oxygen emission spectra were subsequently recorded and integrated.

Plots of integrated singlet oxygen emission intensity versus absorbance were constructed, and the slopes of the resulting linear fits were used to calculate singlet oxygen quantum yields according to Equation (12):

(12) $$\Phi_{x}=\Phi_{st}\frac{Grad_{x}}{Grad_{st}}$$

where Grad_x_ and Grad_st_ are the slopes obtained for the investigated compound and the reference standard, respectively.

## 4. Conclusions

The present study addresses a fundamental question in thiopurine photochemistry: what determines the efficiency of singlet oxygen generation in systems that already exhibit near-unity quantum yield of triplet-state formation? Classical thiopurines, such as 6TG and 6TGuo, efficiently populate the T_1_ state, yet their performance as photosensitisers remains limited. This apparent contradiction suggests that the key factor governing their photochemical behaviour lies not in the formation of the triplet state, but in its subsequent fate. By introducing a tricyclic etheno moiety into the thiopurine scaffold, we have demonstrated that the deactivation pathways of the T_1_ state can be systematically altered without significantly affecting its formation. The investigated analogues retain the characteristic features of thiocarbonyl systems, including efficient intersystem crossing and microsecond lifetimes of triplet states, yet exhibit markedly different reactivity.

In particular, lowering the triplet state T_1_ energy and modifying the excited state landscape create more favourable conditions for interaction with molecular oxygen.

A central finding of this work is that the efficiency of singlet oxygen generation is governed by the degree to which the T_1_ population is directed towards productive pathways. This is reflected in the higher S_Δ_ values observed for the tricyclic analogues, indicating that a larger fraction of the T_1_ population participates in energy transfer to oxygen, resulting in singlet oxygen generation. In contrast, in canonical thiopurines, a substantial portion of the triplet-state population is lost to competing nonproductive processes.

Importantly, our previous studies have shown that, in the absence of oxygen, the triplet states of tricyclic thiopurine analogues are not quenched by naturally occurring nucleosides [32]. The absence of this deactivation pathway is a critical distinction from canonical thiopurines, where interactions with nucleic acid components significantly limit triplet-state availability. As a result, the tricyclic analogues maintain a larger effective population of reactive triplet states under biologically relevant conditions. These combined effects translate directly into enhanced singlet oxygen generation. Despite comparable efficiencies of triplet formation, the tricyclic analogues produce singlet oxygen with significantly higher quantum yields than 6TG and 6TGuo. This demonstrates that structural modification of the thiopurine scaffold enables control over the balance between competing deactivation pathways, effectively redirecting the photochemical outcome.

## Figures and Tables

**Table 1:** Quantum yields and rate constants of the deactivation processes of the T_1_ excited state of TEGuo and 6MeTEGuo in deoxygenated aqueous and acetonitrile solutions at room temperature.

	TEGuo		6MeTEGuo	
	ACN	H_2_O	ACN	H_2_O
E_T_ [cm^−1^] ^a^	*18,010*		*18,080*	
$\Phi_{p}^{0}$ ^b^	<1 × 10^−4^	<1 × 10^−4^	<1 × 10^−4^	<1 × 10^−4^
$\Phi_{ISC}$ ^c^	0.97	0.82	0.91	0.87
$\Phi_{R}$ ^d^	<10^−4^	<10^−4^	<10^−4^	<10^−4^
$\Phi_{nr}$ ^e^	0.97	0.82	0.91	0.87
$\varepsilon_{T}$ [M^−1^ cm^−1^] ^f^	4540 (680 nm)	5140 (600 nm)	4025 (670 nm)	4960 (620 nm)
$\tau_{T}^{0}$ [μs] ^g^	2.05	1.05	1.82	0.83
$k_{0}$ [s^−1^] ^h^	4.8 × 10^5^	9.5 × 10^5^	5.3 × 10^5^	1.1 × 10^6^
$k_{sq}$ [M^−1^ s^−1^] ^i^	4.61 × 10^9^	2.87 × 10^9^	1.02 × 10^9^	4.2 × 10^9^
${\Sigma k}_{nr}$ [s^−1^] ^j^	4.81 × 10^5^	9.52 × 10^5^	5.49 × 10^5^	1.20 × 10^6^
$k_{p}$ [s^−1^] ^k^	<54	<116	<62	<145

^a^ E_T_—the energy of the T_1_ state determined from the phosphorescence spectrum in glassy matrix (MeOH:CH_2_Cl_2_ 1:1) at 77 K; ^b^ room-temperature phosphorescence quantum yield at infinite dilution, estimated to be <10^−4^, which is the sensitivity limit of our instrument; ^c^ quantum yield of triplet-state formation; ^d^ quantum yield of photochemical decay of the compounds (c = 1.6 × 10^−4^ M) in the absence of oxygen ^e^ quantum yield of nonradiative processes; ^f^ molar triplet–triplet absorption coefficient; ^g^ concentration independent, intrinsic lifetime of the triplet state ($\tau_{T}^{0})$; ^h^ concentration independent decay rate constant of the triplet state; ^i^ self-quenching rate constant; ^j^ rate constant of non-radiative processes; ^k^ rate constant for radiative processes.

**Table 2:** Photophysical Parameters of Singlet Oxygen Generation.

	TEGuo		6MeTEGuo		6TGuo	
	ACN	H_2_O	ACN	H_2_O	ACN	H_2_O
$\tau_{T}$ [ns]	131	526	118	449	95	382
k_q_ [M^−1^s^−1^]	3.7 × 10^9^	3.2 × 10^9^	4.1 × 10^9^	3.8 × 10^9^	5.1 × 10^9^	4.2 × 10^9^
Φ_∆_ (air)	0.56	0.28 *	0.54	0.27 *	0.27	0.15 *
S_∆_	0.62	-	0.67	-	0.32	-

*—value determined in D_2_O,$\;\tau_{T}$—lifetime of the triplet-state T_1_ in the presence of oxygen for a compound concentration c = 1.3 × 10^−5^ M; k_q_—rate constant for the quenching of the triplet state by O_2_; Φ_∆_—quantum yield of singlet oxygen sensitisation in a solution saturated with air; S_∆_—the fraction of the triplet state population quenched by dissolved oxygen, participating in singlet oxygen generation.

## Data Availability

The original contributions presented in this study are included in the article/Supplementary Materials. Further inquiries can be directed to the corresponding author.

## Supplementary Materials

The following supporting information can be downloaded at https://www.mdpi.com/article/10.3390/ijms27125482/s1.
