# Supplementary material for: Modulation of Triplet-State Reactivity and Enhanced Singlet Oxygen Generation in Tricyclic Thiopurine Analogues
Source: Int J Mol Sci. 2026 Jun 17;27(12):5482. doi: 10.3390/ijms27125482 (PMC13300233; doi:10.3390/ijms27125482)
Supplement: Supplementary file 1 [file ijms-27-05482-s001.zip › ijms-4385466-supplementary.pdf]

Electronic Supporting Information

Modulation of Triplet-State Reactivity and Enhanced Singlet Oxygen Generation in Tricyclic Thiopurine Analogues

Katarzyna Taras-Goslinska [1,*], Katarzyna Krancewicz [1] and Bronislaw Marciniak [1,2]

[1] Faculty of Chemistry, Adam Mickiewicz University, Uniwersytetu Poznanskiego 8, 61-614 Poznan, Poland; katarzyna.krancewicz@amu.edu.pl (K.K.); marcinia@amu.edu.pl (B.M.)
[2] Centre for Advanced Technology, Adam Mickiewicz University, Uniwersytetu Poznanskiego 10, 61-614 Poznan, Poland

* Correspondence: karem@amu.edu.pl

Content:

1. **Table S1.** Quantum yields and rate constants of the deactivation processes of the T_1 excited state of TEGua and 6MeTEGua in deoxygenated aqueous and acetonitrile solutions at room temperature

2. **Table S2.** Photophysical Parameters of Singlet Oxygen Generation

3. **Figure S1.** Singlet oxygen emission spectra sensitized by 6TG, 6TGuo, TEGua, and TEGuo, recorded in air-equilibrated acetonitrile.

Table S1. Quantum yields and rate constants of the deactivation processes of the T_1 excited state of TEGua and 6MeTEGua in deoxygenated aqueous and acetonitrile solutions at room temperature

	**TEGua**		**6-Me-TEGua**	
	**ACN**	**H_2O**	**ACN**	**H_2O**
**E_T [cm^{-1}]**[a]	18 870		18 800	
Φ_P^0 [b]	<1.0 × 10^{-4}	<1.0 × 10^{-4}	<1.0 × 10^{-4}	<1.0 × 10^{-4}
Φ_{ISC} [c]	0.98	0.87	0.93	0.90
Φ_P [d]	< 10^{-4}	< 10^{-4}	< 10^{-4}	< 10^{-4}
Φ_{nr} [e]	0.98	0.87	0.93	0.90
**ε_T [M^{-1} cm^{-1}]**[f]	4520 (680 nm)	5150 (610 nm)	3975 (680 nm)	4910 (620 nm)
**τ_T^0 [µs]** [g]	2.81	1.50	2.70	1.26
**k_0 [s^{-1}]** [h]	3.56 × 10^5	6.67 × 10^5	3.70 × 10^5	7.94 × 10^6
**k_{sq} [M^{-1} s^{-1}]**[i]	4.01 × 10^9	2.76 × 10^9	3.90 × 10^9	2.61 × 10^9
**k_{nr} [s^{-1}]** [j]	3.56 × 10^5	6.67 × 10^5	3.70 × 10^5	7.94 × 10^5
**k_P [s^{-1}]**[k]	< 0.37 × 10^2	< 0.77 × 10^2	< 0. 40 × 10^2	< 0.89 × 10^2

a) E_T – the energy of the T_1 state determined from the phosphorescence spectrum in glassy matrix (MeOH:CH_2Cl_2 1:1) at 77 K; b) room-temperature phosphorescence quantum yield at infinite dilution, estimated to be < 10^{-4}, which is the sensitivity limit of our instrument; c) quantum yield of triplet state formation; d) quantum yield of photochemical decay of the compounds (c = 1.6 × 10^{-4} M) in the absence of oxygen; e) quantum yield of nonradiative processes; f)molar triplet-triplet absorption coefficient; g) concentration independent, intrinsic lifetime of the triplet state; h) concentration independent decay rate constant of the triplet state; i) self-quenching rate constant; j) rate constant of non-radiative processes; k) rate constant for radiative processes

Table S2. Photophysical Parameters of Singlet Oxygen Generation

	TEGua		6MeTEGua	
	ACN	H_2O	ACN	H_2O
**τ_T [ns]**	64	379	57	327
**k_q [M^{-1} s^{-1}]**	8.00×10^9	7.04×10^9	8.95×10^9	8.15×10^9
**Φ_Δ (powietrze)**	0.61	0.31*	0.61	0.31*
**S_Δ**	0.64	-	0.67	-

* – value determined in D_2O, τ_T – lifetime of the triplet state T_1in the presence of oxygen for a compound concentration c = 1.3 $\times 10^{-5}$M; k_q– rate constant for the quenching of the triplet state by O_2; Φ_Δ – quantum yield of singlet oxygen sensitisation in a solution saturated with air; S_Δ – the fraction of the triplet state population quenched by dissolved oxygen, participating in singlet oxygen generation

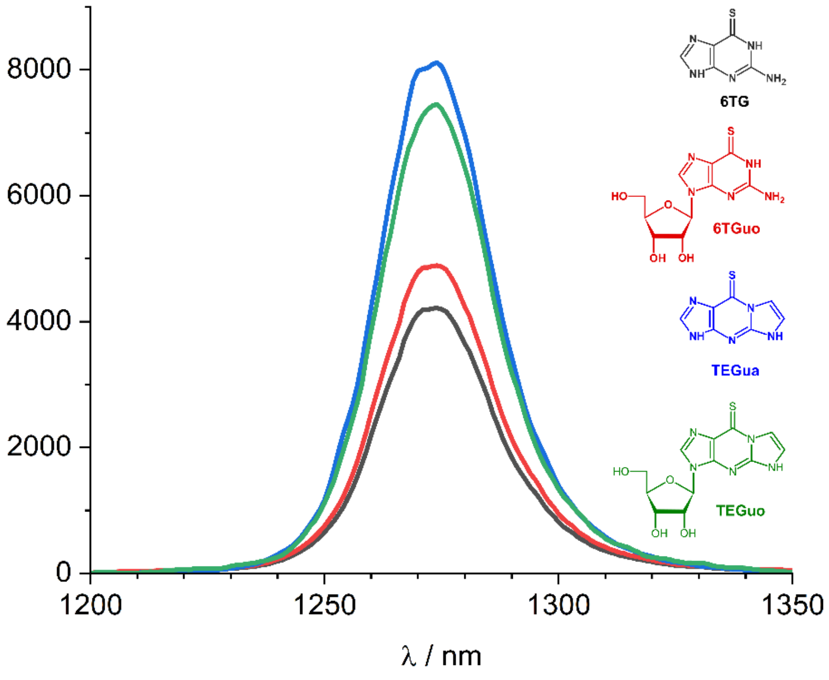

Figure S1. Singlet oxygen emission spectra sensitized by 6TG, 6TGuo, TEGua, and TEGuo, recorded in air-equilibrated acetonitrile. Spectra were recorded at $\lambda_{em} \approx 1270$ nm following excitation at λ_{exc} = 350 nm. The absorbance of each sample at the excitation wavelength was matched (A_{350} = 0.35), and identical experimental conditions were maintained throughout the measurements.
